# Case Series: Therapeutic Combination of VoluDerm Radiofrequency Microneedling and Glycolic Acid Peel in Scaled-Up Concentrations

**DOI:** 10.1093/asjof/ojae075

**Published:** 2024-09-10

**Authors:** Alex Levenberg, Yuri Vinshtok, Amikam Gershonowitz

## Abstract

**Background:**

Glycolic acid (GA) is an α-hydroxy peeling agent that causes controlled removal of the epidermis, with or without the dermis. Studies have shown the ability of GA to stimulate fibroblast proliferation, induce collagen synthesis, and decrease collagen degradation. The VoluDerm radiofrequency microneedling (RFMN; Pollogen, Tel Aviv, Israel) utilizes an array of microelectrodes to penetrate the epidermis and deliver energy to the skin. The controlled fractional thermal injury promotes neocollagenesis in the correction of skin laxity and wrinkle reduction. It was theorized that GA and VoluDerm could synergistically boost collagen production in combined treatment.

**Objectives:**

Safety and efficacy of the combination treatment were investigated in patients seeking corrections of the age-related skin deteriorations.

**Methods:**

Seven female patients (aged 42-70 years, Fitzpatrick II-IV) with photo- and chrono-damaged skin received 5 treatments of VoluDerm followed by the GA peel at increasing 30% to 70% concentrations.

**Results:**

Clinical photography taken 6 months after the treatment demonstrated improvement in elasticity, wrinkling, roughness, pigmentation, erythema, and pore size across the entire treated group. Efficacy quantified by the physician and patients per 1 to 5 Global Aesthetic Improvement Scale was average 4.3 and 4.5, respectively. The treatments were tolerated well without preprocedural anesthesia. Skin phenomena observed after GA application were suggestive of the acid passing to deeper layers.

**Conclusions:**

A novel combination of VoluDerm RFMN and GA at increased concentrations provided safe and effective synergy in the treatment of aging facial skin. Visible results demonstrated skin tightening, reduction of rhytidids, and improvement of the skin texture which may be a result of the combination.

**Level of Evidence: 4:**

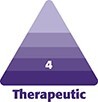

Glycolic acid (GA) belongs to the group of α-hydroxy acids, the chemical peeling agents, which cause controlled removal of the epidermis, with or without the dermis. Studies have shown ability of GA to reverse the cutaneous aging by stimulating fibroblast proliferation, inducing collagen synthesis, and decreasing collagen degradation.^1-3^

Outcomes of GA peel are determined by the acid pH and the time of exposure to the skin cells. For practical use, pH corresponds to the GA concentration. In concentrations <30%, GA cannot bypass stratum corneum (SC) and the peeling effect is associated with the ability to reduce adhesion and cohesiveness of the epidermal corneocytes resulted in desquamation.^4^ At these low strengths, the peel has been used for temporary smoothing the skin and fine wrinkles, reducing solar keratoses, and lightening solar lentigines, but is incapable of affecting deep wrinkles or deep pigmentations.^5^ At higher concentrations (50%-70%), GA already demonstrates the ability to penetrate through the epidermal/dermal junction. In an in vitro study, Jiang and Qureshi^1^ evidenced the presence of GA in the dermis after applying 40% to 60% GA on fresh human skin samples. The authors concluded that GA at high concentration and low pH “may have disturbed the SC and may result in damage to the skin barrier properties.” Song et al^6^ demonstrated the reduction of the skin barrier manifested by increased transepidermal water loss after application of 50% GA to the forearm skin for 3 min. However, the penetration is not uniformed and leads to uneven absorption of the acid in the skin, resulting in focal dermonecrosis and crusting.^7-9^

Radiofrequency microneedling (RFMN) utilizes an array of microneedle electrodes to deliver radiofrequency (RF) current to the dermal layers. The microneedles forcefully penetrate SC and follow with RF emission to create a thermal injury to the dermis. The impact generates fractional zones of thermal ablation and surrounded coagulation, which promote neocollagenesis in the correction of skin laxity, texture, and wrinkle reduction.^10^

Contrary to the bleeding and pain associated with mechanical penetration in many RFMN devices, VoluDerm RFMN (Divine Pro, Pollogen, Tel Aviv, Israel) uses RF to pass through the epidermis in a miniscule hole and without complications. RF is emitted over the entire noninsulated microneedle length and produces a high volume of heat in the dermis. The magnitude of therapeutic impact is determined by the power and duration of the RF pulses. The degree of coagulation is proportioned to the pulse duration, whereas the depth of the ablation channel is determined by the RF power.^11^

We theorized that GA and VoluDerm could synergistically boost collagen production if combined in the treatment application. As the initial step, safety and efficacy of the combination were investigated in a small group seeking corrections of the age-related skin deteriorations.

## METHODS

Collection and management of the patient data were conducted in accordance with the ethical guidelines and principles of the 1975 Declaration of Helsinki. A signed informed consent for the treatment procedures and publishing (including images and data) was obtained from all participants in the current investigation prior to the treatments. Adult female patients with uneven skin texture, mild-to-deep rhytids, and hyperpigmented spots volunteered for the combination treatments in the outpatient clinic of a certified plastic surgeon (A.L.) between March 2022 and February 2023. Pregnant females and patients with keloidal tendency, active acne, or chronic dermal conditions were excluded.

The treatment course included 5 sessions spaced 2 weeks apart. Concentration of GA was progressively increased at each subsequent session, from the initial 30% to 70%, in increments of 10%. The scale-up was intended to gradually deepen the acid impact.^3^ Topical GA applied biweekly in scaled-up (35%-70%) concentrations previously demonstrated to be safe and caused only transient erythema and burning sensation.^12^

### Treatment Procedures

The procedure started with VoluDerm treatment (Video). VoluDerm's microneedle matrix generates 36 entry points over 1 cm^2^ skin area at each pass. The entries achieved by the RF-assisted penetration mechanism warranted minimal treatment discomfort. Settings for the pulse power were gradually increased to tolerable maximum to ensure a high ablation depth. The pulse time was set at medium duration to avoid overcoagulation. An entire face was treated with a single pass, while the most problematic areas (deep wrinkles, solar lentils, and prominent skin laxity) received 2 passes. The second stacking pass was applied to the same initial spot with the VoluDerm tip rotated 1-quarter clockwise. The “quarter-turn” maneuver increased the density of the stimulation impact without missing the spot.

Immediately after VoluDerm, GA solution was applied over the face in brisk dispersing movements. An instant erythema and patterned micro-edema appeared on the skin and were gradually accompanied by whitish “fog” and the light “frosting.” A flow of cooled air was used to relieve the stinging and burning sensation. After 7 min, GA acidity was neutralized with plenty of cold water. The discomfort was relieved, and the frosting and erythema subsided. The treatment procedure was completed with topical 0.67% trolamine emollient to hydrate the skin and shorten downtime. Patients were advised to use topical moisturizer and sun protection for 1 week thereafter.

### Assessment Measures

Clinical photographs were taken at baseline and 6 months after the last treatment. Condition of the facial skin was quantified according to the photo-numeric Scientific Assessment Scale of Skin Quality (SASSQ) parameters: elasticity, wrinkles, skin surface roughness, pigmentation, erythema, and pore size^13^ (Table 1). Pre- and posttreatment measures were compared with the draw conclusion of the changes. Photographic changes were independently graded by the treating physician and patients according to the Global Aesthetic Improvement Scale (GAIS): (5) very much improved, (4) much improved, (3) improved, (2) unchanged, and (1) worsened. Procedural discomfort was assessed separately for RFMN and GA, using the 1 to 10 Visual Assessment Scale (VAS).

**Table 1.** Scientific Assessment Scale of Skin Quality^13^

Parameter	Intensity scale				
	0, none	1, mild	2, moderate	3, severe	4, very severe
Elasticity	No loss of elasticity	Slight loss of elasticity	Moderate loss of elasticity	Severe loss of elasticity	Very severe loss of elasticity
Wrinkles	No wrinkles	Slight wrinkles	Moderate wrinkles	Sever wrinkles	Very severe wrinkles
Pigmentation	No uneven pigmentation	Slight uneven pigmentation	Moderate uneven pigmentation	Severe uneven pigmentation	Very severe uneven pigmentation
Erythema	No erythema	Slight erythema	Moderate erythema	Severe erythema	Very severe erythema
Pore size	0, fine	1, small	2, moderate	3, large	4, very large
	Fine pore size	Small pore size	Moderate pore size	Large pore size	Very large pore size

Participants interviewed 6 months after the original treatment, subjectively reported satisfaction with the treatment results as (1) dissatisfied, (2) undecided, and (3) satisfied.

## RESULTS

**Figure 1.**
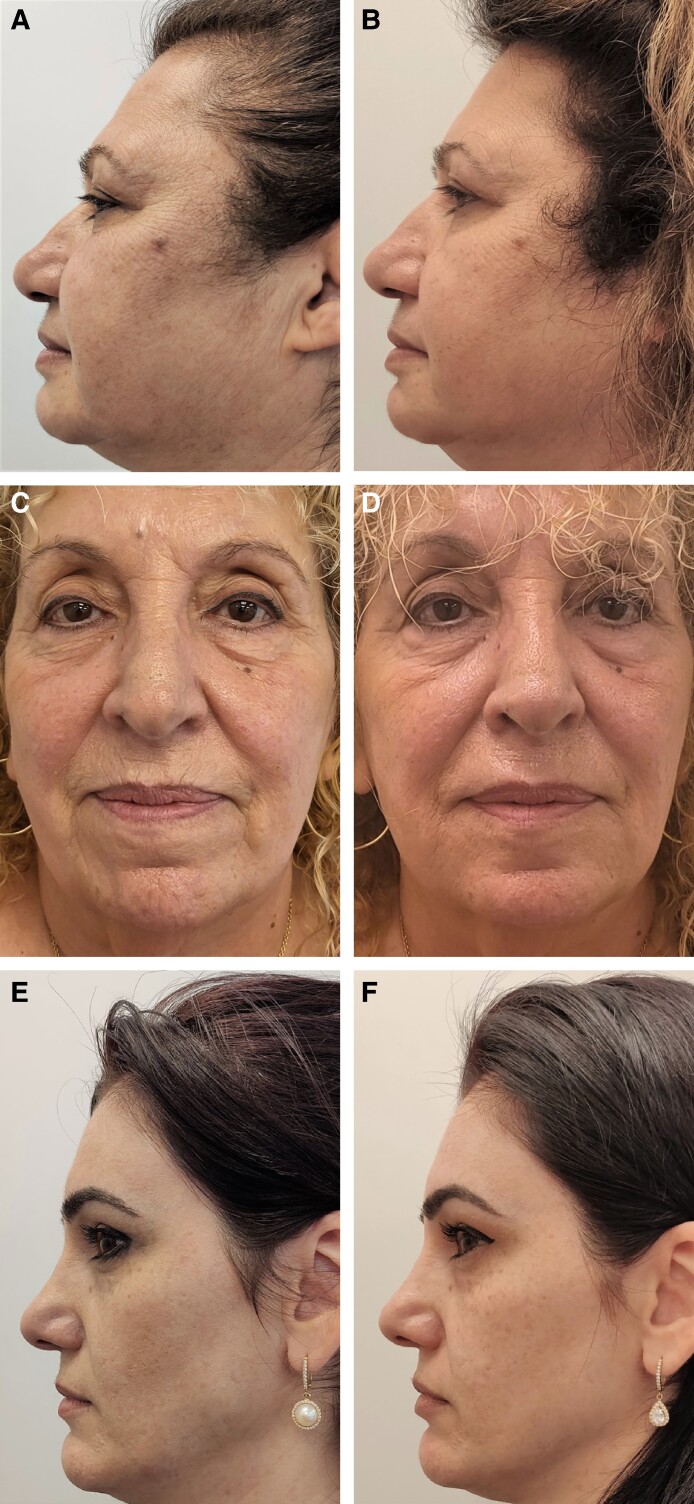
Before and after photographs indicate skin tightening, reduced wrinkles, and improved skin texture 6 months after the treatment: a 62-year-old patient before (A) and after (B), a 72-year-old patient before (C) and after (D), a 42-year-old patient before (E) and after (F).

Seven patients (age range, 42-70 years, Fitzpatrick range II-IV) with photo- and chrono-damaged skin received the treatment course (Table 2). Six months thereafter, patients reported self-perceived smoothening and tightening of the skin, more defined jawline, and less pigmentation (Figure 1). SASSQ indicated improvement in all categories across the treated group: elasticity decreased by average 40%, wrinkling—by 47%, skin roughness—by 55%, pigmentation—by 38%, erythema—by 33%, and pore size—by 47%. Photo-based changes were quantified by the physician and the patients at average GAIS of 4.3 and 4.5, respectively. Patients were satisfied with the achieved results at the 6-month follow-up, with a mean satisfaction score of 2.57.

**Table 2.** Patient Demographics and Skin Quality Assessment (SASSQ)^a^

	Age	Fitzpatrick skin type	Elasticity before/after	Wrinkles before/after	Skin roughness before/after	Pigmentation before/after	Erythema before/after	Pore size before/after
1	69	II	4/2	4/2	4/2	3/2	0/0	4/2
2	70	II	4/2	4/2	4/2	3/2	1/1	4/2
3	41	IV	1/1	2/1	2/1	4/2	0/0	2/1
4	52	III	2/1	3/1	3/1	2/1	0/0	1/1
5	60	III	2/1	2/1	3/1	3/2	0/0	2/1
6	40	II	1/1	1/1	2	3/2	0/0	2/0
7	54	II	1/1	1/1	2/1	3/2	2/1	3/1

^a^0 = none; 1 = mild; 2 = moderate; 3 = severe; 4 = very severe.

The treatments were tolerated well without preprocedural anesthesia. The RFMN discomfort was mild, at an average VAS of 4.4. The GA sensation intensified with an increase in the acid concentration and reached an average VAS of 7.6. Cold air was sufficient to relieve the skin irritation and patient's anxiety.

## DISCUSSION

To our knowledge, this is the first trial implementing a novelty combination of RFMN and GA at increased concentrations. Clinical photographs taken 6 months after the treatment course demonstrated not only significant skin resurfacing, but also effective skin tightening and wrinkle smoothing effects. It was assumed that RFMN and GA generated synergistic thermal and chemical impacts that would activate neocollagenesis toward enhanced skin regeneration.

Kim and Won^14^ showed increased fibroblast proliferation and collagen production in response to GA in a dose dependent manner in vitro. In a histology study, Narda et al^15^ demonstrated that low-to-medium concentration GA caused desquamation and increased collagen levels by 6% with 8% to 15% GA and by 10.1% with 25% GA.

**Figure 2.**
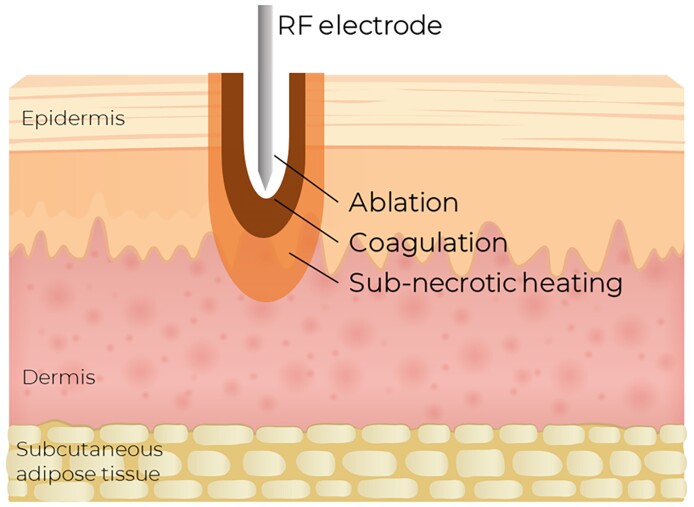
Thermal impacts of noninsulated VoluDerm radiofrequency microneedling to the skin: (1) ablation channel, (2) surrounding zone of coagulated tissue, and (3) volumetric area of subnecrotic heating.

Accordingly, RFMN thermal impact triggers the wound-healing mechanism to regenerate the degrading extracellular matrix.^16^ The high temperature generated by RF emission evaporates the tissue along the entire microneedle length and creates a microscopic cone-shaped ablation channel surrounded by the zones of coagulation and volumetric heating, triggering the surrounding viable tissue to induce the biological effect (Figure 2). Gershonowitz and Gat^17^ reviewed histological skin samples taken during the 2-week period after VoluDerm RFMN. An immediate coagulative necrosis in the epidermis and in the papillary and upper reticular dermis completely resolved to dermal renewal and epidermal regeneration on Day 14. The healing was accompanied by a significant increase of the epidermal mitotic index and collagen content, as quantified in an ex vivo human skin model.^18^ Shapiro^19^ evaluated VoluDerm for reduction of facial wrinkles in 37 patients. The photographic analysis revealed reduction in the Fitzpatrick wrinkle score in 97% of cases, along with significant volume enhancement, decrease of nasolabial folds and periorbital lines, and lessening of the skin laxity. The authors related the achieved efficacy to the process of collagen remodeling that continued 3 months after the last treatment. The safety profile of VoluDerm treatments causes low discomfort, does not require anesthesia, and is associated with less to no downtime.^16,19^

The impact is structurally similar to the micro-channels created by fractional ablative lasers. The laser photo-thermolysis causes columns of the ablation and coagulation zones at the epidermal/dermal level surrounded by viable tissue.^20^ The ablation channels were found effective and safe for so-called laser-assisted drug delivery (LADD) in the treatment of melasma, rhytids, and keloids.^21^ The channels are thought to overcome the epidermal barrier and serve as drug reservoirs for further diffusion. Hydrophilic liquid materials with low molecular weight were found readily absorbed and diffused through the coagulation zone.^22^ Rationally, the hydrophilic nature, low molecular weight (76.05 g/mol), and high water solubility (0.1 g/mL) provide GA with highly potential for diffusion from the VoluDerm ablation channel.

Artzi et al^23^ speculated that the micro-wounds created by RFMN could deepen propagation of the peeling agent and thereby lead to better results. In his comparative study, the RFMN combined with immediate 20% trichloroacetic acid (TCA) was shown to improve skin dyschromia, laxity, and wrinkling at higher rate than TCA peeling alone. However, a longer downtime was reported with the combination treatments.

**Figure 3.**
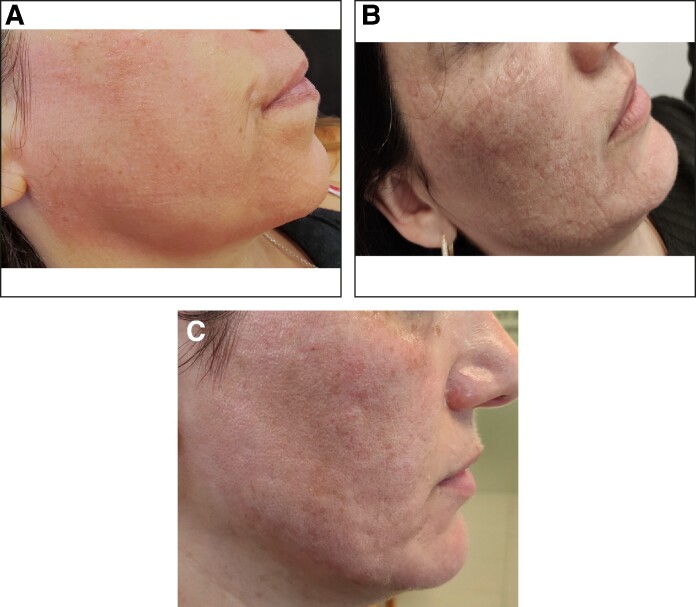
Skin reaction after combination application of VoluDerm radiofrequency microneedling and glycolic acid in a 42-year-old female patient: (A) immediate micro-edema, erythema, and whitened haze; (B) subsequent skin frosting; and (C) reduced erythema and frosting after the acid neutralization.

In our investigation, we pretreated the skin with VoluDerm RFMN before applying GA peel at increased concentrations. We assumed that VoluDerm could facilitate transdermal propagation of GA by disrupting the SC barrier. Based on the sequential appearance of the skin erythema, edema, fogging, and light frosting, it was concluded that GA could gradually penetrate deeper than it would without RFMN (Figure 3). Generalized erythema was indicative of the acid diffused in the epidermis.^4^ The appearance of micro-edema was due to GA filling the ablation channels and triggering a reactive release of the extracellular fluid. Exposure of the tissue to glycolic acidity led to the peptide denaturation, manifested as whitened haze and light frosting. As an indicator of exfoliation into the dermis, frosting is not achieved with GA at <50% concentration and is usually associated with strong peels like TCA, which has a well-known ability for deep penetration.^4^

### Safety Aspects

No serious or systemic events that would require therapy were identified. Procedural bleeding usually expected with RFMN was absent as noninsulated VoluDerm microneedles emitted RF along the entire length and cauterized dermal micro-vessels in the ablated surface.

Although intradermal dispersion of glycolic acidity may raise safety concerns, current treatments produce only transient sequalae of erythema, edema, fogging, and frosting that subsided after the acid neutralization. We took into consideration 2 factors determining the degree of the acid impact on the skin—its intensity (directly related to the acid concentration) and duration of the acid exposure to the skin. Accordingly, we optimized the protocol by limiting the highest GA concentration to 70% and by restricting its exposure to 7 min. Due to its hydrophilic nature, GA activity was sufficiently neutralized by washing it off with large amounts of water.^24^

This is a preliminary report of the novelty treatment approach; therefore, the investigation is limited by the sample size and lack of the control group. Although the safety of the treatments was demonstrated, further investigation is warranted to verify the reproducibility of the effectiveness and clarify the best treatment protocol. Although skin improvement succeeded, further optimization of the VoluDerm parameters would be beneficial. Fine tuning of the pulse duration should provide a degree of coagulation that would not block diffusion of the acid and, at the same time, be sufficient for triggering the wound-healing cascade.

## CONCLUSIONS

A novel combination of RFMN and GA at medium-to-high concentrations provides safe synergy in the treatment of aging facial skin.
